# WiSPH: A Wireless Sensor Network-Based Home Care Monitoring System

**DOI:** 10.3390/s140407096

**Published:** 2014-04-22

**Authors:** Pedro Magaña-Espinoza, Raúl Aquino-Santos, Néstor Cárdenas-Benítez, José Aguilar-Velasco, César Buenrostro-Segura, Arthur Edwards-Block, Aldo Medina-Cass

**Affiliations:** 1 College of Telematics, University of Colima, Avenida Universidad 333, C.P. 28045 Colima, Col., Mexico; E-Mails: aquinor@ucol.mx (R.A.-S.); arted@ucol.mx (A.E.-B.); 2 Department of Innovation and Technological Development, Siteldi Solutions S.A. de C.V., 111 Canario Street, C.P. 28017 Colima, Col., Mexico; E-Mails: nestor.cardenas@siteldisolutions.mx (N.C.-B.); jose.aguilar@siteldisolutions.mx (J.A.-V.); cesar.buenrostro@corporativostr.com (C.B.-S.); aldo.medina@corporativostr.com (A.M.-C.)

**Keywords:** wireless sensor networks, WiSPH, home care monitoring systems, fall detection, e-Health

## Abstract

This paper presents a system based on WSN technology capable of monitoring heart rate and the rate of motion of seniors within their homes. The system is capable of remotely alerting specialists, caretakers or family members via a smartphone of rapid physiological changes due to falls, tachycardia or bradycardia. This work was carried out using our workgroup's WiSe platform, which we previously developed for use in WSNs. The proposed WSN architecture is flexible, allowing for greater scalability to better allow event-based monitoring. The architecture also provides security mechanisms to assure that the monitored and/or stored data can only be accessed by authorized individuals or devices. The aforementioned characteristics provide the network versatility and solidity required for use in health applications.

## 1. Introduction

### 1.1. Research Background and Motivation

Globally, an aging population provides a good indicator of how health services have progressed in both developed and developing countries. However, to better provide these benefits, a series of challenges must first be met. One extremely important challenge is to train much needed healthcare professionals who specialize in providing care to seniors who often suffer from a variety of chronic diseases associated with aging, and design environments that incorporate wireless technologies and communications systems adapted to the needs of the geriatric community [1]. Projections show that between 2000 and 2050 the number of people above the age of 60 will increase from 11% to 22% worldwide, meaning that persons in this age group will number approximately 2 billion [2].

Aging presents a series of challenges for the entire world population, primarily because seniors slowly lose their ability to be self-sufficient due to chronic diseases, physical and/or mental disabilities, or the general frailty that characterizes the aging process [2]. Any of these conditions represent factors that limit the elderly or endanger their lives, even within the confines of their homes. Consequently, 24-hour-a-day monitoring of the elderly can improve attention provided for chronic or acute health concerns, accidents such as falls, as well as a series of other conditions that can detrimentally affect the elderly. For example, falls represent the second most common cause of death by accident among the aged, making persons over the age of 60 the most vulnerable population group. Additionally, non-fatal falls by the elderly can severely compromise quality of life and/or represent considerable medical expenditures (*i.e.*, in Finland $3,611 dollars per injury, in Australia $1,049 dollars per injury) [3].

Providing remote healthcare monitoring and services presents a series of important challenges; therefore, it is important to generate remote monitoring strategies to provide primary healthcare services and mechanisms that allow seniors to receive long-term assistance. To better meet the needs of the aging population, research has significantly advanced both the theory and application of e-Health technologies; largely because their application can reduce costs generated by patient monitoring and provide a variety of advanced services [4]. Importantly, studies show that the elderly generally accept e-Health technologies and consider them beneficial [5].

### 1.2. Research Objective

The main objective of this research was to design, develop and implement a system (WiSPH) in conjunction with IEEE 802.15.4 to detect and alert trained professionals about persons falling to the ground, as well as event-based monitoring to report tachycardia and bradycardia [3]. The objectives of this work include:

1. developing a fall detection algorithm to handle data transmitted from an accelerometer;
2. generating an event-based, reliable and scalable network algorithm;
3. employing a reliable data encryption scheme (AES) for use within the WSN;
4. creating a domestic WSN infrastructure which allows patients freedom of movement without losing communication;
5. programming a mobile application and Web page for easy access to live monitoring and information access;
6. transmitting *push notifications* of abnormal events to be relayed to caretakers, doctors or family members.

## 2. Related Work

### 2.1. E-Health Applications

E-Health applications are gaining popularity and greater acceptance because of their versatility and reduction of care-taking costs. Nowadays diverse systems and health-centered applications are being developed, which according to their application can be categorized in: daily living activities, fall and movement detection, location tracking, medication intake and medical status monitoring [6]. Applications for daily living activities focus on monitoring the activities of individuals within a predetermined space. One important example of this type of project is *AICO*, which utilizes a sophisticated combined Bayesian network that includes the input from a series of environmental parameters that can create an approximation of the activities being carried out by an individual [7]. Another application of this kind is *Caregiver's Assistant*, which employs RFID cards and a database that includes 38,000 human activities and a fast inference mechanism that allows persons to identify the actions of others remotely within a given space [8].

Applications that focus on fall and movement detection focus on following user movements and detecting falls. One example of this kind of service is *Smart HCN*, which consists of a WSN that monitors the posture of the subject and images taken by cameras to alert a specialist if the subject has suffered an accident [9]. Another example of an application that focuses on fall and movement detection is presented in work done by [10], which uses an accelerometer located at head-height to transmit data that can detect a fall and send a notification to a mobile personal digital assistant.

Location tracking applications are based mainly on the principle of identifying the location of users and analyzing their behavior. One example of this kind of application is presented in work done by [11]. This system employs a WSN to obtain RSSI values, which, through an algorithm, can locate the location of users within their homes. *ZUPS* is also an application that centers on location tracking for aged and/or disabled people. This project uses a ZigBee network and ultrasound-positioning systems, which allows caregivers to not only locate individuals within a specified space, but to also provide assistance for persons moving from one place to another beyond the confines of their home [12].

Medication intake applications consist mainly in monitoring the intake of the patient's drugs. *iCabiNET* provides a solution that employs a smart medicine manager that can notify patients via SMS or audio alarms at home to remind patients about their medications, as well as dosages and times [13]. Another medication intake application by the name of *iPackage* consists of medication wrappers with RFID tags which can be detected by an RFID sensor at the moment of ingestion, allowing caregivers to remotely monitor whether or not a patient is adhering to instructions [14].

Finally, medical status monitoring collects clinical variables (*i.e.*, heart rate, glucose monitoring, pulse, *etc.*), elaborates a current-state diagnosis of the patient and provides the information to caretakers. If any abnormality is detected, it can be immediately communicated to either family members or caretakers. *AlarmNet* is an application that monitors a series of physiological variables, storing the data and processing the information to detect any abnormalities. If an abnormality is found, it notifies a mobile assistant [15]. *Baby Glove* is an application that monitors vital signs of newborn babies, such as their body temperature. The data is gathered through the baby's romper and then transmitted to a WSN, which constantly supervises important physiological variables and notifies caregivers if there is reading beyond the programmed limits in real time [16].

Although there are many e-Health applications available today, this work focuses on developing a hybrid application that focuses on fall and movement detection and on medical status monitoring in a controlled environment, based on a WSN, to detect falls, tachycardia and bradycardia for the elderly population. To achieve this, this research focused on the use of an accelerometer and a heart rate sensor, connected to a previously developed mobile monitoring node to collect data and send it to the WSN Infrastructure, only when abnormal readings are detected.

### 2.2. E-Health Platforms

E-Health applications can have different classifications, depending on their specific objective (fall detection, activity monitoring, localization, *etc.*). Several authors [17,18] have identified a series of requirements for healthcare applications that are based on wireless technologies, including:

1. Reliability: the transmission of precise data, which involves preventing the duplication of information, by implementing an efficient Quality of Service (QoS) which insures a high Packet Delivery Ratio (PDR) and reduces the Packet Loss Rate (PLR).
2. Energetic efficiency: the development or use of an energy-saving algorithm or technology to reduce the consumption of energy in devices, because this helps extend the life span of the network.
3. Data routing: the use of an efficient communication protocol that provides scalability, failure tolerance and best possible route selection, among others.
4. Node mobility: the ability of free moving wireless nodes to move within an indoor area, always maintaining optimum connectivity.
5. Security: the use of efficient security mechanisms to protect the data and privacy of highly sensible patient information, including its robbery or corruption.

Taking into account the proposed requirements for the development of a robust e-Health, Table 1 shows a comparative among the developed systems and platforms. Considering the requirements an e-Health solution, and taking into account the characteristics already existing platforms, our workgroup selected WiSe [19] (primarily because of its low energy consumption) with the goal of developing a robust, reliable system that integrates the best characteristics of the platforms/systems depicted in Table 1.

## 3. Proposal Architecture

**Figure 1.**
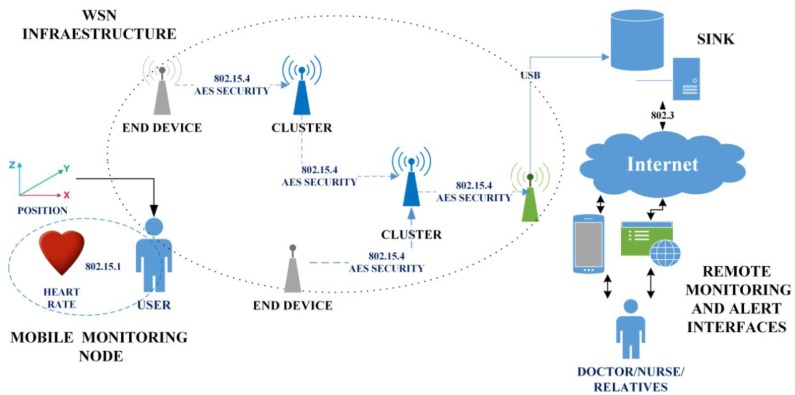
The proposed system architecture.

This section describes the proposed network architecture. Figure 1 illustrates the general system. The proposed system architecture consists of: (a) Mobile monitoring node; (b) WSN Infrastructure; (c) SINK; (d) Remote monitoring and alert interfaces.

### 3.1. Architecture

The mobile monitoring node receives, processes and forwards the data pertaining to tachycardia, bradycardia and falls throughout the networks. The WSN Infrastructure consists of a group of nodes placed throughout the home. These nodes are capable of establishing a hierarchical network, receiving information from the mobile nodes and routing the information to a computer with reduced computational capabilities and an 802.15.4 radio. The SINK is a computational device that picks up and stores the information it receives from the WSN infrastructure, permitting either local or remote access to the data. Finally, the Remote Monitoring and Alert Interface is an application to serve mobile devices and a standard website, both of which can receive notifications of important events and consult the patient's history and other personal information.

### 3.2. Mobile Monitoring Node

**Figure 2.**
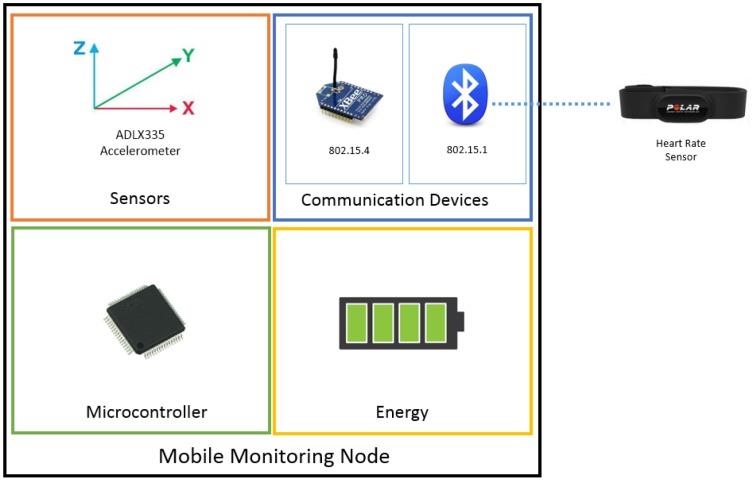
Block diagram of the Mobile Monitoring Node.

The mobile monitoring node consists of an LCP2148 ARM7 micro controller, a radio that is 802.15.4 compatible and a radio that is 802.15.1 compatible, an energy module and a pair of sensors (accelerometer and heart rate sensor). The node is programmed to constantly monitor the values of the heart rate sensor and the accelerometer to detect abnormal values which could indicate an event that may compromise the user's safety. Figure 2 illustrates the Mobile Monitoring Node.

The triple axis accelerometer contained in the mobile device is used to detect rapid accelerations. These sensors are normally fabricated with small margins of error, which can be compensated for by means of software [25]. To calibrate each axis of the accelerometer, it is necessary to apply a different compensation value (offset) for each axis, (*a_x_* = 1 g, *a_y_* = 0 g, *a_z_* = 0 g), (*a_x_* = 0 g, *a_y_* = 1 g, *a_z_* = 0 g), (*a_x_* = 0 g, *a_y_* = 0 g, *a_z_* = 1 g). In order to deliver these samples, the micro-controller's Analogical to Digital Converter (ADC) is configured to 4.5 MHz with a 10-bit resolution, which obtains the 3 axes at a 10 ms frequency and stores them. In order to calculate the offset, the X, Y and Z-axes were fixed at 0 g. The value of the ADC in the Y-axis when the acceleration is equal to 0 g is 505; the value of the ADC in the X-axis when the value is 0 g is of 510; the value of the ADC in the Z-axis with a 0 g acceleration is 515. Consequently, the three-axes of the accelerometer and the ADC values of each axis can be adjusted using the following formulas.

The calibration formula for the Y-axis is:

(1) $$a_{y}=\frac{\left(\frac{ADC_{y}\times 3.3}{1024}\right)-1.63}{0.33}$$

The calibration formula for the X-axis is:

(2) $$a_{x}=\frac{\left(\frac{ADC_{x}\times 3.3}{1024}\right)-1.64}{0.33}$$

The calibration formula for the Z-axis is:

(3) $$a_{z}=\frac{\left(\frac{ADC_{z}\times 3.3}{1024}\right)-1.65}{0.33}$$

The Signal Magnitude Vector (SMV) was calculated to detect a possible fall, which, according to [26], is SMV > 1.8 g.

The formula to calculate the SMV is:

(4) $$\text{SMV}=\sqrt{a_{x}^{2}+a_{y}^{2}+a_{z}^{2}}$$

**Figure 3.**
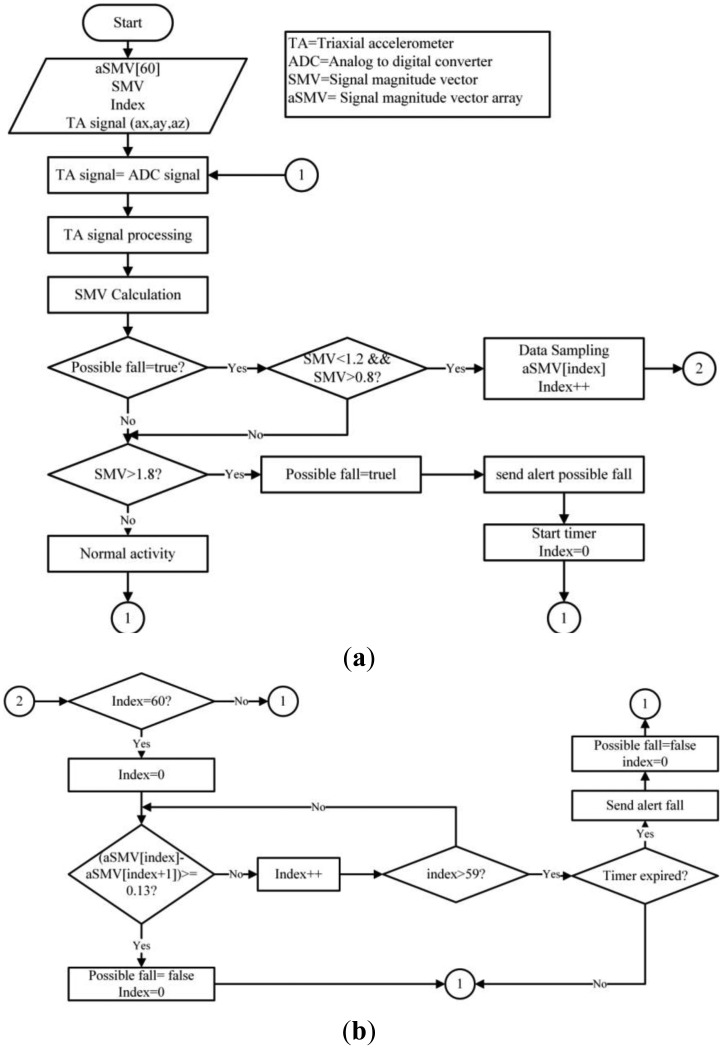
(**a**) Fall detection algorithm (part 1); (**b**) Fall detection algorithm (part 2).

Once a possible fall has been detected, the PNR algorithm determines if a fall has actually occurred. The PNR algorithm is shown in Figure 3. The PNR algorithm activates when the SMV readings average 1 g to determine if the individual is on the floor and hurt due to an accident before it transmits a distress signal. Wang's algorithm, [10] which was used to validate falls, uses a sample rate of 60 SMV entries and compares each incoming entry with the 59 preceding samples. If the product of these samples is not above or the same as 0.13 g, a value that is easily surpassed if the user stands up or walks normally, an alert will be sent to the network.

To detect abnormalities in the user's heart rate, a monitoring threshold was established based on beats per minute (BPM) measurements. One common heart rate abnormality is bradycardia, which is lower than 60 BPM for individuals at rest, although this condition may vary with age and daily habits of the individual. Older people, in particular, have bradycardia if their value drops below 50 BPM because the heart weakens and the heartbeat slows as persons age [27].

Another common heart beat abnormality is tachycardia. Adults with tachycardia have over 100 BPM [28]. As with bradycardia, a series of habits and age influence the values; however, these factors do not require in-depth study. Based on other research, the acceptable limits for tachycardia and bradycardia were set at 100 BPM and 60 BPM, respectively. Any values above or below the established limits would activate the alarm.

### 3.3. WSN Infrastructure

The network used for the detection and transmission of data was developed using WiSe nodes; therefore, the nodes have a low energy consumption rate [19]. Low energy consumption is a very important consideration because the network life, integrity and security for health applications requires exceptional reliability [17].For these reasons, the WSN infrastructure employs a 128-bit AES encryption algorithm to protect network integrity and security, ensuring that the information is not intercepted by nodes that do not belong to the network [29].

A hybrid routing protocol that is dynamic, scalable, destination-centered, with a hierarchical structure was developed for the network. The system is based on IEEE 802.15.4, which contemplates the use of mobile nodes that form the network infrastructure. The infrastructure nodes handle the Received Signal Strength Indication (RSSI) to proactively create the network and can serve as: Cluster Head (CH), End Devices (ED) and SINK. On the other hand, the mobile node only sends data when an event occurs (either and increased or decreased heart rate, or a fall). Therefore, the information is sent to a single network node that immediately reacts to the alert and finds the event destination. To cover this and other functions, the proposed routing algorithm uses a series of network packets which are described in Table 2.

To create the network's structure, the nodes begin in an undefined state, except for the SINK. Each node periodically sends HELLO packets to localize any possible neighboring nodes. The HELLO packets are then broadcast and received by all of the immediate neighbors and is not retransmitted, this to prevent network flooding. Once the undefined nodes receive an answer (HACK packet) from a defined structure node (CH, ED or SINK), it will change its status (ED) and will stop transmitting the HELLO packets; then it will select the best link based on the Received Signal Strength Indication (RSSI). The functions of the nodes (ED) are to send DATA packets from a mobile node to its respective (CH) and reply to the HELLO packets received from undefined nodes. When a node (ED) is connected to another infrastructure node, it creates and stores its new neighbor in a routing table; afterwards, it assumes the role (CH). The (CH) then routes the information received from the (ED and mobile nodes), maintaining the network nodes connected and replying to HELLO packets to the undefined infrastructure nodes. On the other hand, mobile nodes are already defined before joining the network. The primary purpose of a mobile node is to monitor user variables, discover the destination of the information monitored for each event and send DATA packets to the network.

**Figure 4.**
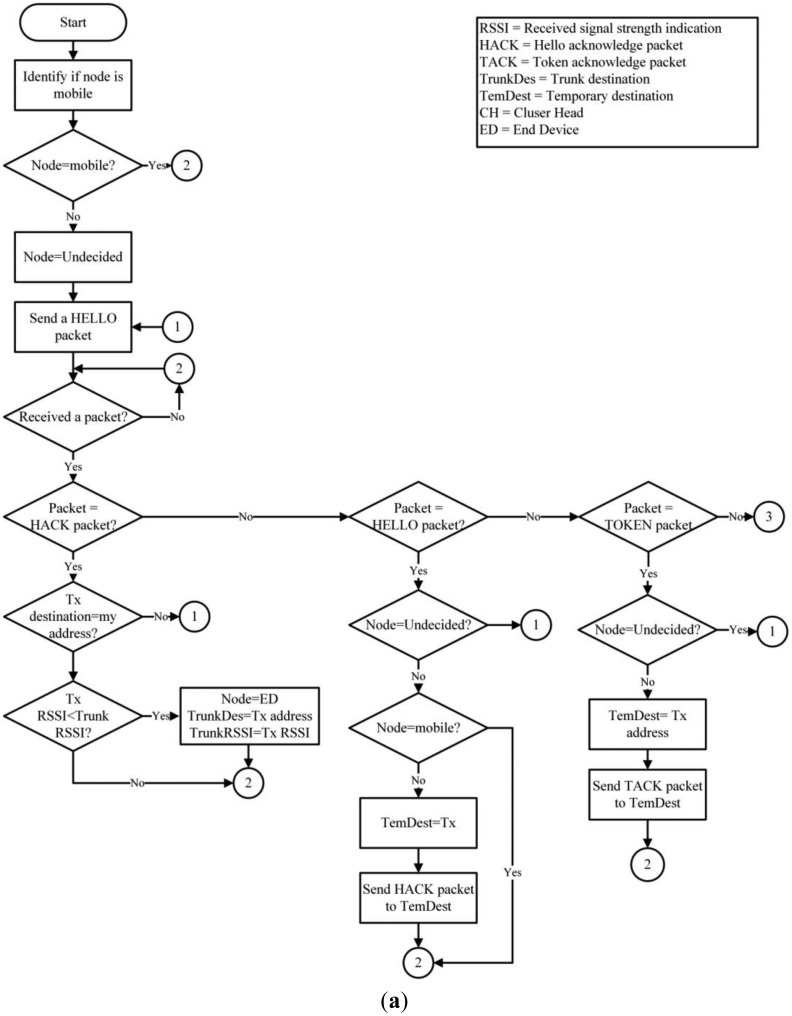

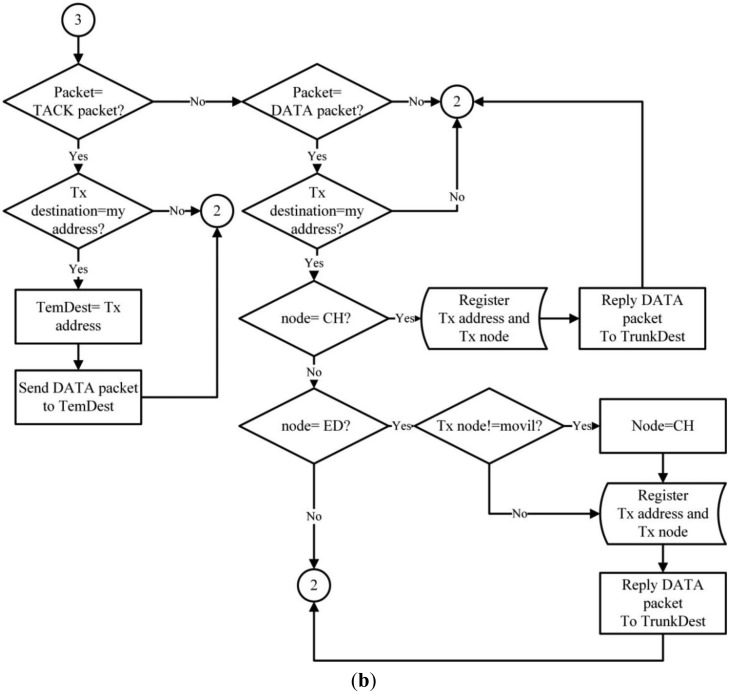
(**a**) Routing Algorithm (part 1); (**b**) Routing Algorithm (part 2).

The routing of DATA packets is carried out by a hybrid mechanism. A mobile node must first reactively identify its destination by broadcasting a TOKEN packet. Only upon receiving a (TACK) unicast reply from a network node will the mobile node send a return DATA package. Importantly, the DATA routing in infrastructure nodes is proactive because each node knows its route by default because the information is kept and actualized in its routing table. Figure 4 illustrates the proposed routing protocol for WSN.

### 3.4. SINK

**Figure 5.**
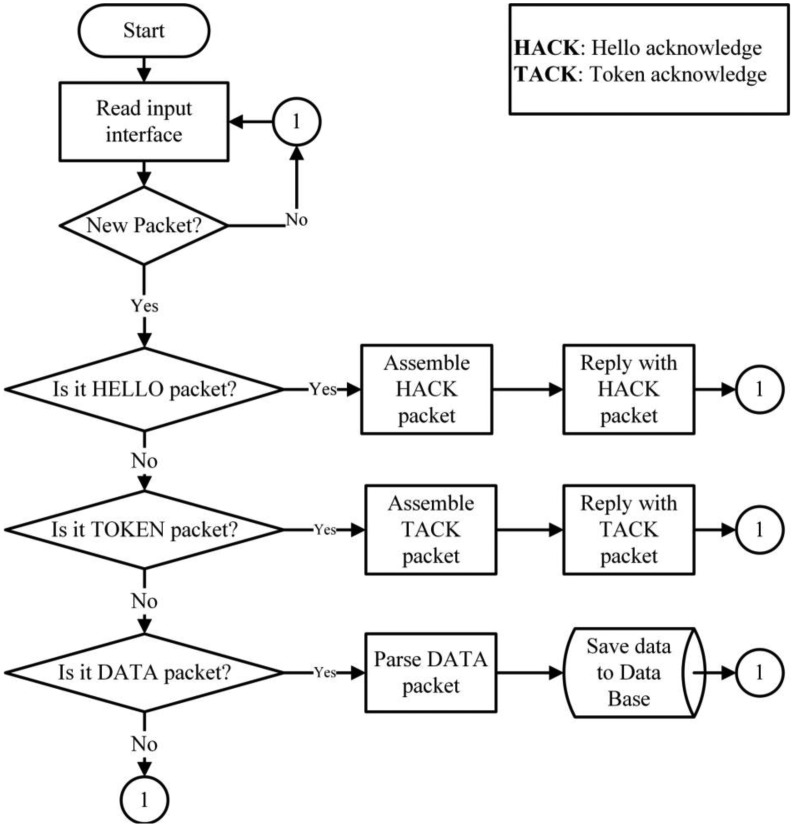
SINK processing algorithm.

The SINK is comprised of electronic devices that have a greater processing capacity than the rest of the devices of the WSN Infrastructure, because it is where all the information compiled by the WSN is sent [30]. The SINK not only collects data, but communicates it through the Internet to the application that controls the database. The behavior of the data collector is shown in Figure 5. The behavior of the SINK is completely reactive as it waits for the arrival of any packet to be processed. When a new package is received, the type of packet must be analyzed. If it is a HELLO packet, the SINK creates a reply HACK packet, which contains information about the packet type, its origin (SINK ID), its destination (obtained from the transmitting node) and its role (role of the SINK). If the SINK receives a TOKEN packet, it sends a TACK packet to the transmitting node. If the SINK receives a DATA packet, it analyzes and classifies the packet content in order to store it in its database.

### 3.5. Remote Monitoring and Alert Interface

**Figure 6.**
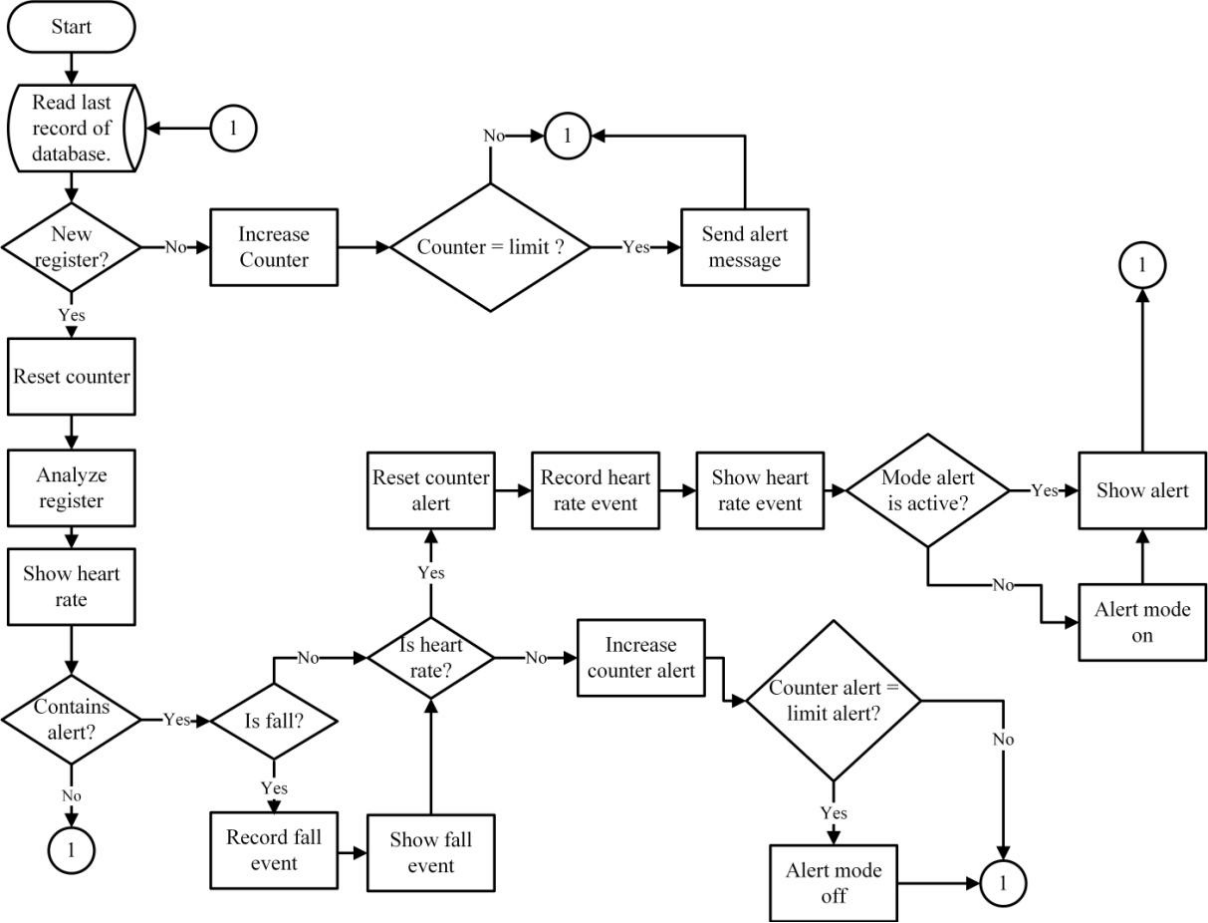
Web interface and alerts flow chart.

The web monitoring system consists of a front-end application which continually analyses every new entry sent to the database server. The system, in real time, can show a user's heart rate, possible and real falls, and can interpret the information and send notifications to caretakers in case of emergency. Figure 6 illustrates the interface functions.

The monitoring system functions reactively because it must first analyze the status of the database before it determines if the entry it is receiving is new. If the system does not detect any changes, an inactivity counter initializes and will compare the counter's reported values with previously set limits for inactivity. If the inactivity counter reports data that are below the established limit, it reinitializes. Importantly, if the inactivity counter reports are equal to the already established values, the system sends an alert message to the user to report a possible network error, because the system is not receiving new entries. In this way, the user can more easily determine if any problem exists. If the entry is new, the counter will automatically reset the counter and identify all the information to be displayed on the mobile or web interface.

Besides the alerts mentioned previously, the system also sends alerts relative to situations that may place the user's physical integrity, making it possible to notify those who are concerned for the patient's wellbeing about a potential fall or a bradycardia or tachycardia event. The alerts are generated upon analyzing any new entries received and recorded in the database. After receiving a new entry, the application analyzes it and validates the type of registry (fall or heart rate). Upon determining the type of registry, the alert counter resets, activating the alert mode and displaying the existing alert on the mobile device or Web page. After resetting, if there are no new entries registered in the database, the alert counter increases. Once the counter increases, the application compares the alert counter with the pre-established counter limits. If the entries surpass the alert counter limits, the alert mode is deactivated. This is an important safety measure as it prevents repeated and unnecessary alerts, or prolonged periods of system alerts.

## 4. Proof of Concept

As a proof of concept for the proposed architecture, five WiSe nodes and one Mobile Node were deployed in a 14 m × 20 m home, in order to mount a WSN infrastructure and ensure coverage throughout the entire home. A 128-bit AES encrypting mechanism was also used to secure all data transmitted throughout the network.

**Figure 7.**
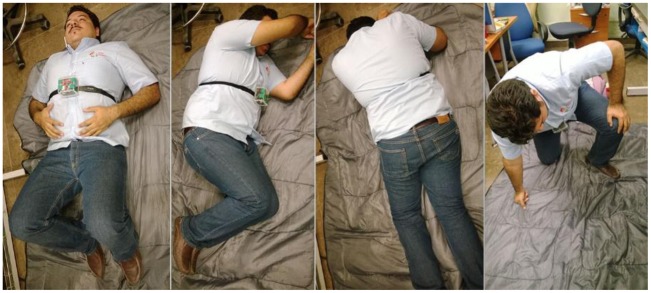
Fall tests.

A total of 40 falls were carried out to validate the fall detection algorithm. The 40 falls were equally represented by 10 falls backwards, 10 falls forward, 10 falls to each side (five falls to the left and five falls to the right) and 10 falls to the knees, as shown in Figure 7.

**Figure 8.**
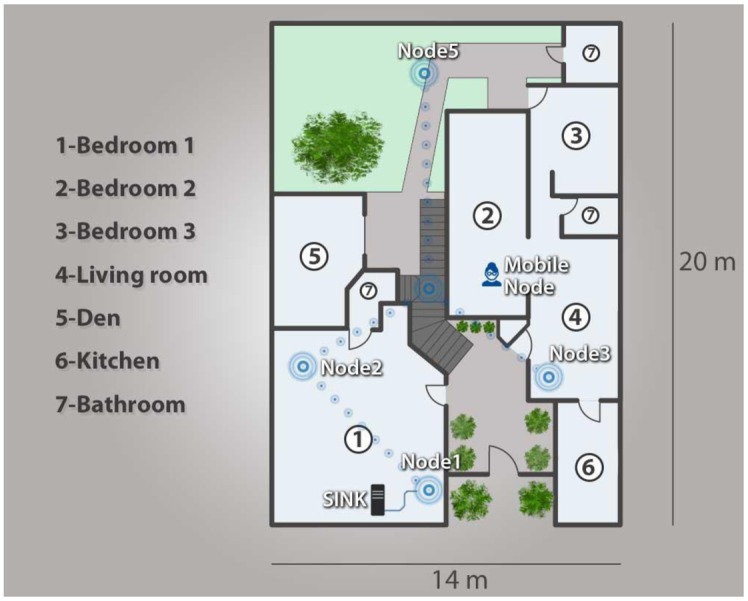
Proof of concept scenario.

A mobile node was programed to send the events (DATA) to the WSN. The monitored data sent to the WSN was also sent to a second computer possessing graph processing software every 500 ms or upon occurrence of an event, which came first. This was done in order to validate the data actually received with the values of the proposed algorithm. To validate the established values for heart rates, 10 stress-related tests were conducted, all of which generated events that were sent throughout the network and stored in the monitoring and alert interface. Figure 8 presents the scenario in which the test was performed.

## 5. Results

This section provides the results of the proof of concept for the WiSPH system, which included:

(a) the PNR algorithm's ability to detect falls;
(b) the software's ability to detect abnormal heart rates;
(c) network metric results (RSSI, packet loss and hops).

### 5.1. Results of the PNR Algorithm for Fall Detection

**Figure 9.**
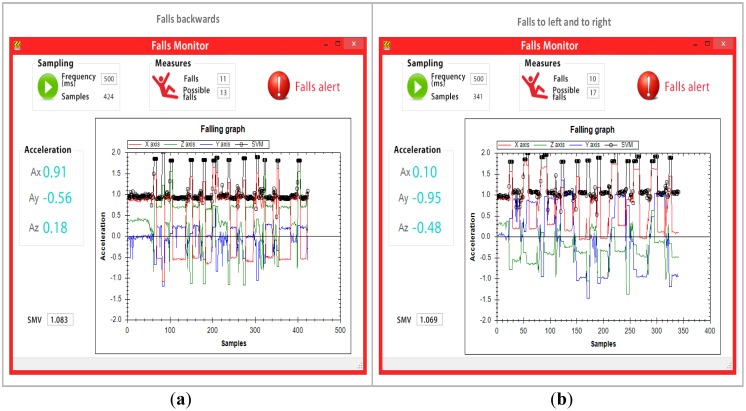

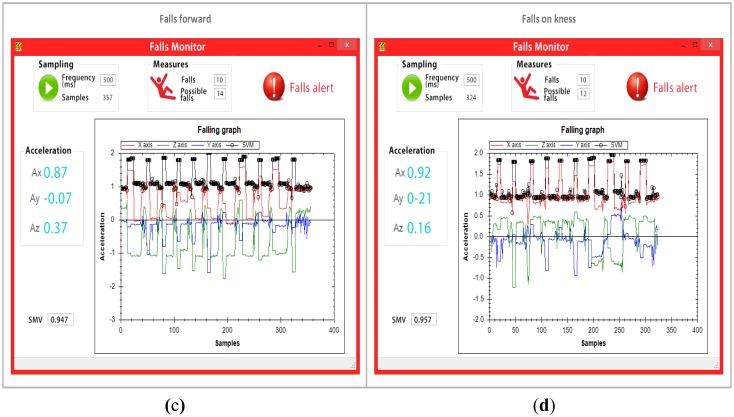
(**a**) Results from backwards falls; (**b**) Results from side falls; (**c**) Results from forward falls; (**d**) Results from knee falls.

Data was transmitted throughout the network and to monitoring software after each event. However, the mobile node only relayed the values provided by the accelerometer to the monitoring software every 500 ms. Figure 9 provides the results obtained from the 40 falls, where the four types of falls (the two types of side falls were categorized as one) captured by the monitoring software are shown. The monitoring software was reset for each kind of fall to assure more precise data. The tests reveal that the mobile node transmitted a total of 1,446 entries for the four types of falls to the monitoring software, which were then processed, analyzed and graphed. The black line in the graph reveals that the SMV remains lower than 1.8 g for normal everyday activities (walking, standing, standing up, sitting down, *etc.*). However, when the user falls, the line rises to levels above 1.8 g, which causes the mobile to alert both the network and the monitoring software of a possible fall. After the simulated fall, subjects remained immobile on the ground, simulating being hurt. This was reported by the high peak of the black line in the graph. Significantly, when the black line does not exceed 0.13 g between entries for more than 30 s the mobile device sends a critical fall alert to both the network and the monitoring software.

In total, 56 possible falls and 41 critical falls were detected in the four tests, meaning that a total of 97 events were created. These events were then relayed across the network to the fall detection software. Only one false alarm occurred, which means that the PNR algorithm identified falls within the margin of error of 2.5%. Table 3 shows the generated DATA packets by falls.

**Figure 10.**
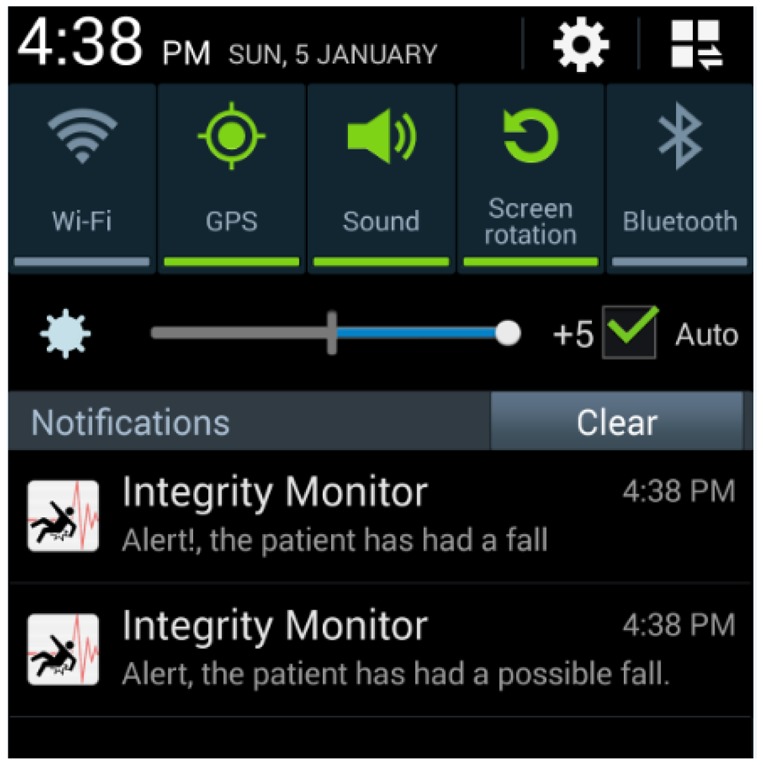
Push notifications from the generated events.

For each possible fall, alerts were generated and delivered to the caregivers by means of the application we developed for mobile devices, via push notifications. Figure 10 illustrates the mobile device interface.

### 5.2. Results from the Detection of Abnormalities in Heart Rate

As was previously mentioned in Section 3.2, [27] and [28] established a monitoring threshold to detect heart rate abnormalities (bradycardia and tachycardia). They established a minimum value of 60 BPM and maximum value of 100 BPM. Based on the abovementioned criteria, we programmed the mobile node to set off an event alarm if it received a value above 100 BPM or 60 BPM, respectively.

**Figure 11.**
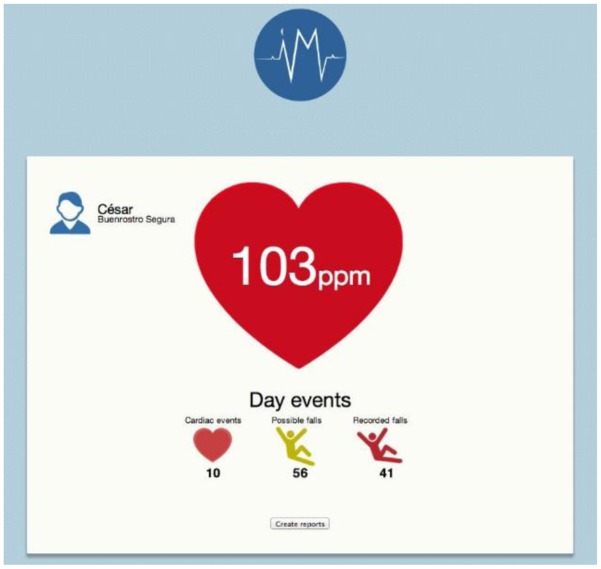
Web System Alert Interface.

Subjects participating in the study were asked to totally relax in order to lower their BPM, establish baselines and to try to activate the alarm. The subjects registered no readings below 60 BMP under normal relaxed circumstances. Unlike, we were able to validate the 100 BPM criteria by having each subject exercise on an elliptical treadmill for 10, 3-minute periods. This permitted the subjects' heart rates to surpass the 100 BPM value, which was consistently achieves as was predicted. Importantly, each time the subjects' heart rates exceeded the 100 BPM limit, the events were transmitted to the network and received by the SINK. The data were then uploaded by the web application to the interface shown in Figure 11.

**Figure 12.**
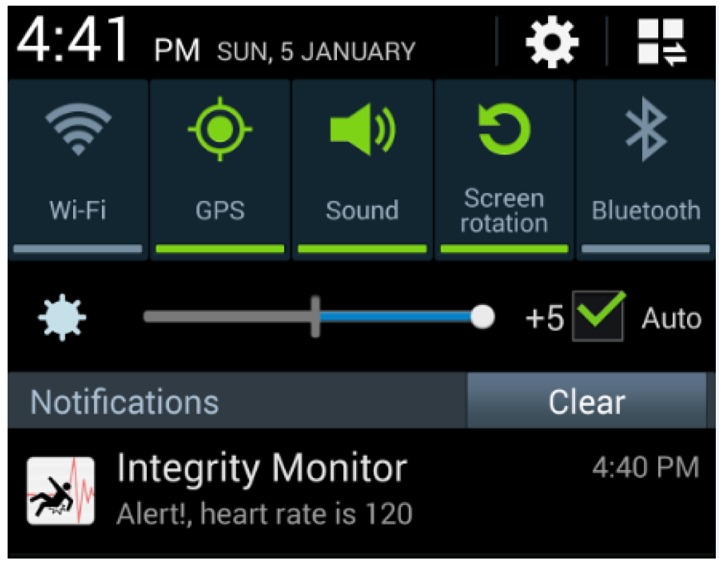
Push notification generated for the heart rate event.

All heart rate event alerts reach the mobile by means of push notifications. These push notifications are then handled by the software application to be visualized. Figure 12 shows the alert notification as viewed on a mobile device.

The results reported in Sections 5.1 and 5.2 involving patient monitoring were carried out under the supervision of the head of the geriatric department of the Institute of Safety and Social Services for State Workers (ISSSTE) of the state of Colima, Mexico.

### 5.3. Network Results

As far as the network is concerned, both the mobile device and the WSN employ a 128-bit AES encrypting mechanism. Two tests were performed to validate the safety device. Two tests were performed to validate the safety device. The first test consisted in introducing an unknown node from an outside network, which in contrast with network nodes, does not have its AES encrypting mechanism enabled. The second test consisted in introducing another unknown node that had an AES key enabled, but which did not correspond to the one used by the WSN. In both cases, the external nodes try to capture HELLO packets transmitted within the WSN. Table 4 shows the structure of packets collected by a network node within the WSN. Each packet begins with an identical byte of information. This is then followed by two bytes to indicate the packet length. The next byte defines the frame type which corresponds to the IEEE 802.15.4 standard (0 × 81-R × 16 indicator), followed by 2 bytes for the source address, 1 byte for the RSSI, and 1 byte for the method sent (0 × 00 for unicast, 0 × 02 for broadcast), which, in this case, is broadcast mode. Finally, each packet contains a 2-byte payload and a 1-byte checksum to verify the integrity of the packet.

**Figure 13.**
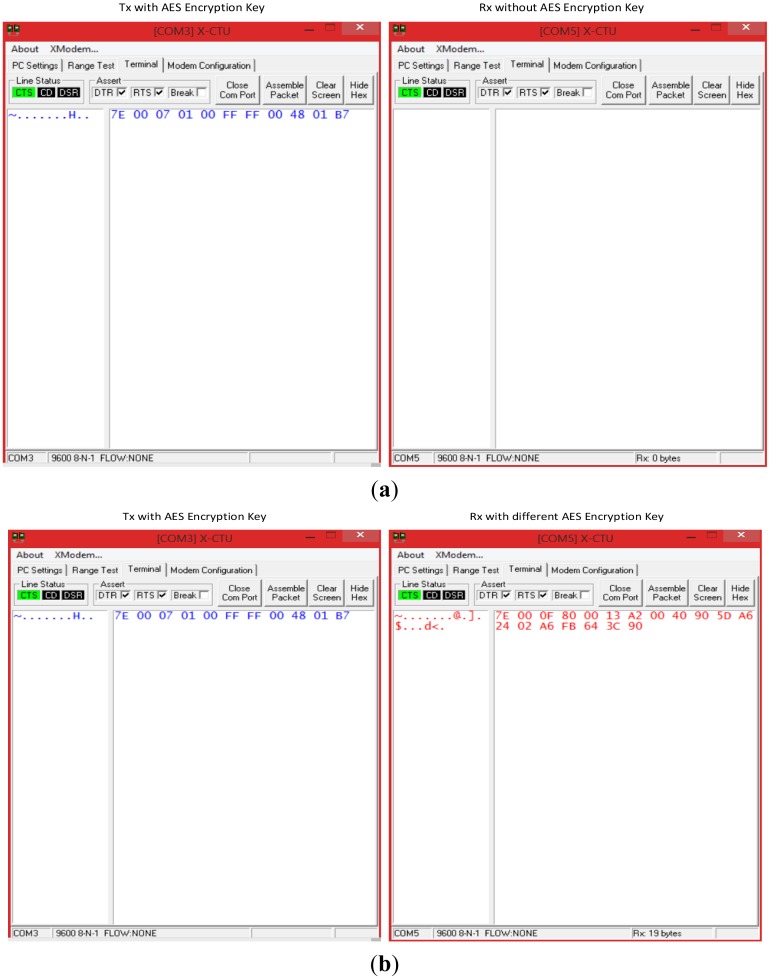
(**a**) Security Test 1; (**b**) Security Test 2.

Figure 13a provides the results of the first security test. Results show that HELLO packets generated by a network node (left terminal) cannot be intercepted (right terminal) because the unknown node does not share the same AES mechanism. Figure 13b provides results that show that an unknown node can receive HELLO packets, but cannot decipher the 11-byte packets as originally sent because they do not possess the same key. An analysis of the packet received by the right terminal external device shows that the first byte is a packet start character (0 × 7E). The following two bytes provide the packet length (0 × 000F), which in this case is 15 bytes, excluding the start bytes, length and checksum. The next byte indicates the frame type, which corresponds to the IEEE 802.15.4 standard 4 (0 × 80) that defines the MAC address used (64 bits). For this reason, the next 8 bytes of the frame contain the physical address of the source node (0 × 0013A20040905DA6). The next byte of the frame indicates the packet's RSSI (0 × 24). The subsequent byte (0 × 02) whether the packet was transmitted by unicast or broadcast, which in this case was broadcast. The following 4 bytes (0 × A6FB643C) provide the packet payload. Lastly, the final byte provides the checksum (0 × 90). A comparison of the original data with the information acquired by the unknown node shows that it could properly get the starting character of the packet, its RSSI, the broadcast mode and the checksum. However, it could not obtain packet information corresponding to the length, frame type, source address and payload correctly. The unknown node cannot acquire this information because it does not possess the network encryption key. Experimental results show, in sum, that critical information regarding network information cannot be successfully obtained by a node that does not form part of the network.

**Figure 14.**
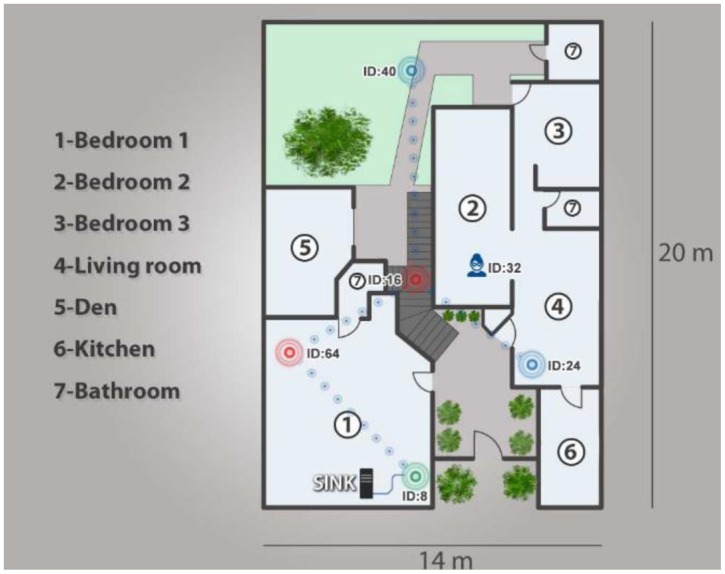
Created network.

Once we tested the security of the WSN network, we proceeded to analyze and validate the functioning of the network. As mentioned previously, five infrastructure nodes were installed in the home to form a network which could communicate remotely to a mobile node, employing the routing protocol our team developed. Figure 14 illustrates the network configuration employed in our proposed network architecture, which employs two Cluster Head devices (ID16 and ID 64), two End Devices (ID 24 and ID 40), a mobile node (ID 32) and a SINK (ID 8).

Once the network is formed, communication between the mobile node and the infrastructure can begin and data can be sent. In this test, a person representing a mobile node moved freely throughout the home to determine the number of hops and RSSI. To do this, data packets were generated and routed to the SINK, as shown in Table 5.

A total of 50 events (40 different falls and 10 tachycardia events) were simulated under test conditions using healthy subjects. Each even generated packets that possessed a sequence number and an event identifier. Adding these values to the DATA packets allowed us to identifying the totality number of packets that were generated and received. From this data set, it was possible to determent the percentage of lost packets and the number of packets corresponding to each event. Specific information is provided by Table 6.

According to the information provided by Table 6, of the 109 DATA packets received, 97 were attributable to fall events, 10 to heart rate events and only two packets were lost, which represents a 1.83% rate of error.

## 6. Conclusions and Future Work

This paper has presented a Home Care Monitoring System (WiSPH), which represents a potentially valuable tool to assist caretakers, family members or health care practitioners to monitor the heart rate and potentially dangerous falls of elderly patients who can still live independent, but assisted lifestyles. The proof of concept testing presented in this work permits us to conclude:

- WiSPH implements a security mechanism (AES) that does not allow nodes from outside the network to decipher data.
- The routing algorithm, based on the IEEE 802.15.4 standard, functions efficiently in health applications as only two of the 109 DATA packets that were generated in this study were lost, representing less than a 1.8% of the packets sent.
- An efficient algorithm was developed to detect falls which correctly reported 100% of serious falls.
- The already established value for tachycardia made it possible to efficiently identify the 10 events that were programmed in this test, resulting in a 100% success rate. In contrast, the value for a low heart rate (bradycardia) could not be validated because subjects could not lower their heart rates below the 70 BPM criteria.
- The monitoring function of the network and alert interface worked well in conjunction. Of a total of 109 push notifications, caretakers 100% of the real-time alerts.

In sum, WiSPH employs important features from various platforms/systems that have been previously compared in this work. WiSPH implements an effective security mechanism and has relatively low power consumption. Our system combines these two characteristics with an efficient fall detection algorithm and an accurate means to detect established thresholds for bradycardia and tachycardia. WiSPH also possesses the flexibility and scalability to add other sensors because the routing algorithm provides the necessary QoS. The abovementioned characteristics not only permit WiSPH to be used by geriatric patients living at home, but with a wide variety of health care scenarios where mobile monitoring of different physiological variables is necessary. In conclusion, the variety of parameters WiSPH effectively monitors and reports on favorably compares to systems that are presently available in the marketplace.

Future work will include in-depth simulations to validate the system performance, in particular to simulate multiple nodes in different spatial areas to confirm its scalability. These simulations will provide the antecedents before performing large-scale real-world implementation inside a regional hospital. Additionally, subsequent work will include optimizing the PNR algorithm to detect different the severity of falls by means of combining enhanced accelerometer data with the accompanying heart rate changes. Another area of work in the future is to optimize the system to include greater personalization according to a patient's specific physiological variables, including amount of nutrition, age, weight, body fat, triglycerides, cholesterol *etc.* Other parameters can include stress test results to set a baseline for elderly patients that can be introduced into the system.

## Figures and Tables

**Table 1.** E-Health Platform Comparison.

**Platforms/Requirements**	**Code Blue [20]**	**MEDiSN [21]**	**MASN [22]**	**KNOWME [23]**	**LOBIN [24]**
**Reliability Mechanism**	None (Unreliable Multicast)	Two-Tier Architecture with Dedicated Wireless Backbone and Optimized Rate Control Protocols	Dynamic Reliability Adaptation Scheme	Multithreading collector	Packet Loss Rate tolerance 2%
**Scheme for Energy Efficiency**	Not Provided	Division of functionality between acquiring and relaying data	Energy-aware cluster formation using energy level determination of sensor nodes	A combination of data buffering, adaptive sensor throttling, and dynamic selection of data transmission methods	Assumes the loss of a small percentage of packets and compensates bandwidth restrictions of the IEEE 802.15.4 technology with a careful design of several communication protocols
**Routing Methodology**	Multicast and multi-hop routing	Many-to-one and one-to-one communication	Intra-Cluster and Inter-Cluster Data Relay	None. Star topology	Dynamic Source Routing
**Techniques for Mobility Support**	Periodic Flooding for Route Discovery	Physiological monitors, periodically select the best relay point to forward their data	None. Does not support real-time data collection under mobility conditions	Nokia N95 capabilities on LAN/MAN (WiFi, 3G, EDGE)	Wireless Communications Infrastructure Subsystem
**Data Security**	Not Provided	128-bits AES encryption	Only the source/destination can decipher the medical data through crypto-keys	End-to-end encryption	Not Specified
**Supported Application**	Medical Care and Disaster Response	Emergency Detection	Real-time collection of ECG Data	General monitoring	Real-time collection And Emergency Detection
**Sensor Scalability**	Not Specified	supports motes with different sensor suites	Yes, it can increase the number of sensors (Does not Specify which)	Not Specified	Not Specified

**Table 2.** Packets generated by the routing protocol.

**Type of Packet**	**Length (Bytes)**	**Description**
HELLO	11	The packet code is 0 × 48. The HELLO packet discovers the network.
HACK	11	The packet code is 0 × 58. The HACK packet confirms reception of the HELLO packet.
TOKEN	11	The packet code is 0 × 54. The TOKEN packet explores the network before sending a DATA packet.
TACK	11	The packet code is 0 × 4 B. The TACK packet confirms that the TOKEN packet was received.
DATA	19	The packet's code is 0 × 44. The DATA packet contains data about the information's origin, as well as the patient's heart rate, the event code, the number of hops and the packet counter. DATA packets, however, can be adapted to carry even more information.

**Table 3.** Generated DATA packets from falls.

**Type of Fall**	**Total of Programed Falls**	**Total of Possible Falls**	**Total of Detected Falls**	**Total of False Alarms**
Back	10	13	11	1
Side	10	17	10	0
Front	10	14	10	0
Knees	10	12	10	0

**Table 4.** HELLO Packet Structure.

**HELLO Packet Structure**										
Begin (1byte)	Length (2bytes)		API (1byte)	Src. Address (2bytes)		RSSI (1byte)	Opt. (1byte)	Payload (2 bytes)		CS (1byte)
0 × 7E	0 × 00	0 × 07	0 × 81	0 × 00	0 × 15	0 × 24	0 × 02	0 × 48	0 × 01	0 × FF

**Table 5.** Scenario routing table.

**ID Node**	**Trunk Destination**	**Hops**	**Link RSSI**
8	D/A	D/A	D/A
16	64	2	−64 dBm
24	16	3	−57 dBm
32	Dynamic	Dynamic	D/A
40	16	3	−47 dBm
64	8	1	−53 dBm

**Table 6.** Received DATA Packets.

**Total Packets**	**Fall DATA Packets**	**Heart Rate DATA Packets**	**Lost Packets**	**Percent of DATA Lost Packets**
109	97	10	2	1.83%

## References

[1] WHO Website 10 Facts on Ageing and the Life Course. http://www.who.int/features/factfiles/ageing/es/index.html.

[2] WHO Website Interesting Facts about Ageing. http://www.who.int/ageing/about/facts/en/index.html.

[3] WHO Website Falls. http://www.who.int/mediacentre/factsheets/fs344/en/index.html.

[4] Boonyarattaphan A., Yan B., Sam C. A security framework for e-health service authentication and e-health data transmission.

[5] Tan O., Wei Kiat K., Jamie N., Wong A., Tay Z., Helander M.G. Are working adults ready to accept e-health at home?.

[6] Alemdar H., Ersoy C. (2010). Wireless sensor networks for healthcare: A survey. Comput. Netw..

[7] Lu C.-H., Fu L.-C. (2009). Robust location-aware activity recognition using wireless sensor network in an attentive home. IEEE Trans. Autom. Sci. Eng..

[8] Philipose M., Consolvo S., Fishkin K., Smith P.I. Fast, detailed inference of diverse daily human activities.

[9] Tabar A.M., Keshavarz A., Aghajan H. (2006). Smart home care network using sensor fusion and distributed vision-based reasoning.

[10] Wang C.-C., Chiang C.-Y., Lin P.-Y., Chou Y.-C., Kuo I.T., Huang C.-N., Chan C.-T. Development of a fall detecting system for the elderly residents.

[11] Yan H., Xu Y., Gidlund M., Nohr R. An experimental study on home-wireless passive positioning.

[12] Marco A., Casas R., Falco J., Gracia H., Artigas J.I., Roy A. (2008). Location-based services for elderly and disabled people. Comput. Commun..

[13] Lopez-Nores M., Pazos-arias J., Garcia-Duque J., Blanco-Fernandez Y. Monitoring medicine intake in the networked home: The icabinet solution.

[14] Pang Z., Chen Q., Zheng L. A pervasive and preventive healthcare solution for medication noncompliance and daily monitoring.

[15] Wood A., Stankovic J.A., Virone G., Selavo L., He Z., Cao Q., Thao D., Wu Y., Fang L., Stoleru R. (2008). Context-aware wireless sensor networks for assisted living and residential monitoring. IEEE Netw..

[16] Baker C.R., Armijo K., Belka S., Benhabib M., Bhargava V., Burkhart N., Der Minassians A., Dervisoglu G., Gutnik L., Haick M.B. Wireless sensor networks for home health care.

[17] Jiménez V.P.G., Armada A.G. (2009). Field measurements and guidelines for the application of wireless sensor networks to the environment and security. Sensors.

[18] Egbogah E.E., Fapojuwo A.O. (2011). A survey of system architecture requirements for health care-based wireless sensor networks. Sensors.

[19] González A., Aquino R., Mata W., Ochoa A., Saldaña P., Edwards A. (2012). Open-wise: A solar powered wireless sensor network platform. Sensors.

[20] Malan D. Codeblue: An *ad hoc* sensor network infrastructure for emergency medical care.

[21] Ko J., Lim J.H., Chen Y., Rvăzvan Musvaloiu-E Z., Terzis A., Masson G.M., Gao T., Destler W., Selavo L., Dutton R.P. (2010). Medisn: Medical emergency detection in sensor networks. ACM Trans. Embed. Comput. Syst..

[22] Hu F., Jiang M., Celentano L., Xiao Y. (2008). Robust medical ad hoc sensor networks (MASN) with wavelet-based ECG data mining. Ad Hoc Netw..

[23] Mitra U., Emken B.A., Sangwon L., Ming L., Rozgic V., Thatte G., Vathsangam H., Zois D., Annavaram M., Narayanan S. (2012). Knowme: A case study in wireless body area sensor network design. IEEE Commun. Mag..

[24] Custodio V., Herrera F., López G., Moreno J. (2012). A review on architectures and communications technologies for wearable health-monitoring systems. Sensors.

[25] Tarng W., Lin C.-H., Liou H.-H. (2012). Applications do wireless sensor networks in fall detection for senior people. Int. J. Comput. Sci. Inf. Technol..

[26] Karantonis D.M., Narayanan M.R., Mathie M., Lovell N.H., Celler B.G. (2006). Implementation of a real-time human movement classifier using a triaxial accelerometer for ambulatory monitoring. IEEE Trans. Inf. Technol. Biomed..

[27] Agruss N.S., Rosin E.Y., Adolph R.J., Fowler N.O. (1972). Significance of chronic sinus bradycardia in elderly people. Circulation.

[28] Budzikowski A., Cho C. Atrial Tachycardia. http://emedicine.medscape.com/article/151456-overview.

[29] Kumar P., Lee H.-J. (2011). Security issues in healthcare applications using wireless medical sensor networks: A survey. Sensors.

[30] Raspberry Pi. http://www.raspberrypi.org/.

